# Stress cardiomyopathy following thyroidectomy in a postmenopausal patient: A case report

**DOI:** 10.1016/j.ijscr.2024.109600

**Published:** 2024-04-04

**Authors:** Margarita Atanasova, Manol Sokolov, Tsvetan Popov, Angel Arabadzhiev, Silvia Ivanova, Gergana Ivanova

**Affiliations:** a Department of Anesthesiology and Intensive Care, University Hospital Alexandrovska, Medical University Sofia, Bulgaria; b Department of Surgery, University Hospital Alexandrovska, Medical University Sofia, Bulgaria; c Department of Cardiology, University Hospital Alexandrovska, Medical University Sofia, Bulgaria; d Medical University Sofia, Faculty of Medicine, Bulgaria

**Keywords:** Stress cardiomyopathy, Thyroidectomy complications, Takotsubo cardiomyopathy

## Abstract

**Introduction and importance:**

First described in 1990 in Japan, Stress cardiomyopathy (SC) is characterized by transient systolic and diastolic left ventricular (LV) dysfunction with a variety of wall-motion abnormalities. It predominantly affects postmenopausal women and is often preceded by an emotional or physical trigger. SC is an increasingly recognized form of transient LV dysfunction that is often completely reversible.

**Case presentation:**

We report a case of SC induced by thyroidectomy in a postmenopausal woman with a good outcome for the patient.

**Clinical discussion:**

The pathogenesis of SC remains obscure, several possible hypotheses include catecholamine induced myocardial spasm or catecholamine related myocardial stunning, metabolic disorders and coronary microvascular damage. It is described as a disease with a 6-fold female–male predominance, affecting elderly postmenopausal women leading researchers to an estrogen-based theory for the pathogenesis. Thera are also increasing evidences for link between SC and thyroid pathology. There is no consensus on the diagnostic criteria for SC.

**Conclusion:**

SC should be kept in mind especially in women with postmenopausal syndrome in perioperative period.

## Highlights

• Stress cardiomyopathy primary affects postmenopausal women, leading to an estrogen-based theory.
• With a 6-fold female predominance, preceded by an emotional or physical trigger
• An increasingly recognized form of LV dysfunction, often completely reversible
• There are increasing evidence to suggest SC in patients with thyroid dysfunction.

## Abbreviations

SC: stress cardiomyopathy
ECG: electrocardiography
CT: computer tomography
POD: postoperative day
LV: left ventricle
EF: ejection fraction
BP: blood pressure
PCI: percutaneous interventions

## 1 Introduction

This case report and applied discussion gives a suggestion of possible reasons of acute cardiomyopathy after thyroid gland surgery. We present the diagnostic process and therapeutic approaches according to international guidelines and our experience.

## 2 Case presentation

**Fig. 1:**
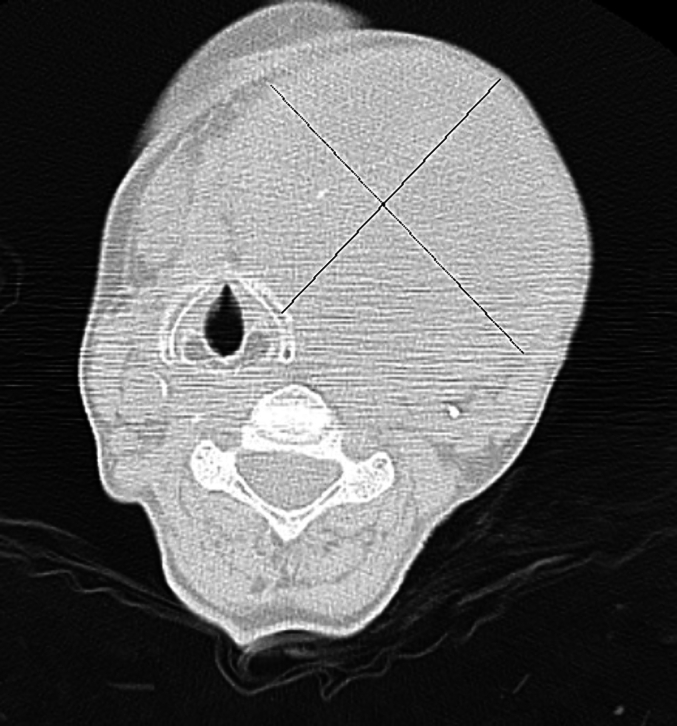
CT of Thyroid Gland.

A 57-year-old woman with no comorbidities was admitted to the Clinic of Surgery due to a growing tumor mass in the thyroid gland for the last year. No symptoms of compression in the cervical region were reported, only weight loss of 5-6kg. Her preoperative examinations were: Asthenic woman, height 162sm, weight 48kg, BMI 18.3; Auscultation revealed normal breathing without wheezing, normal hearth sounds; BP 130/80mmHg, heart rate 100/min, ECG - sinus tachycardia, left position, no registered pathological deflections, no ischemic changes in all leads, chest X-ray - normal, blood tests showed mild anemia: Hb 106g/L. A thyroid function tests were normal: free T3 of 2,9pg/mL, free T4 of 0,93ng/dL, and TSH of 0,72uIU/mL. Local status of neck lesion – thyroid gland enlargement 4th degree, solid consistency, immovable to surrounding structures, mobile when swallow. CT preoperatively: thyroid gland - generally enlarged with a spherical shape, mostly at the expense of the left lobe. Its dimensions reach 93/115/130mm. The cranial part of the gland is visualized dorsolaterally to the left at the C4 level. The caudal part of the gland is established at the level of the upper thoracic aperture, without passing distally to the mediastinum. The gland presents with an inhomogeneous structure. Cystic components are not demarcated. There was a significant mass effect for major vascular structures in the neck region. The formation dislocated left cricoid and thyroid cartilage, with preserved structure. The trachea was compressed and shifted to the right, with a reduction of the lumen. The CT revealed no distant metastatic lesion (Fig. 1). Perioperative risk assessment reported only smoking as a risk factor.

The patient underwent thyroidectomy and lymph node dissection under general anesthesia after informed consent was signed. Intraoperatively 1 unit of blood was transfused due to blood loss of about 800mL. The operation was completed successfully. Hemodynamic fluctuations were not observed intraoperatively and in the early postoperative period. Histological findings revealed anaplastic thyroid carcinoma.

**Fig. 2:**
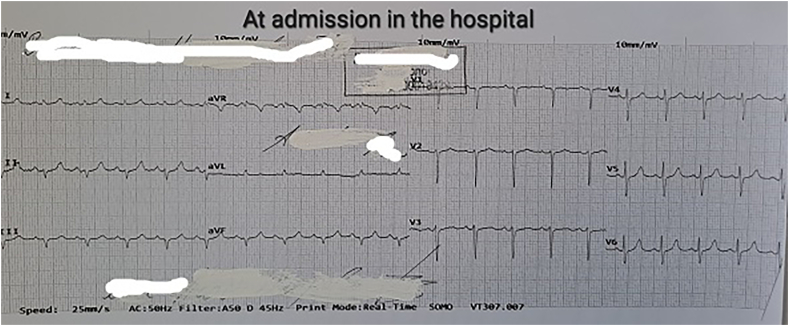
ECG at admission in the hospital.

**Fig. 3:**
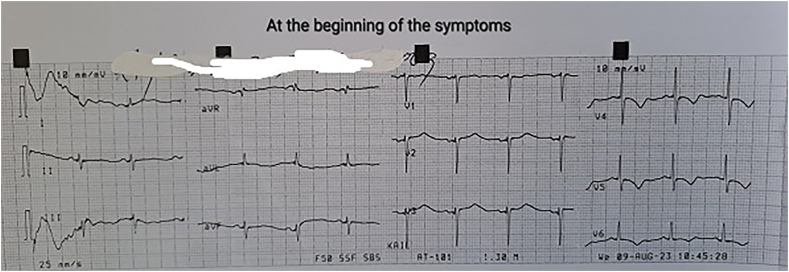
ECG at the beginning of the symptoms.

**Fig. 4:**
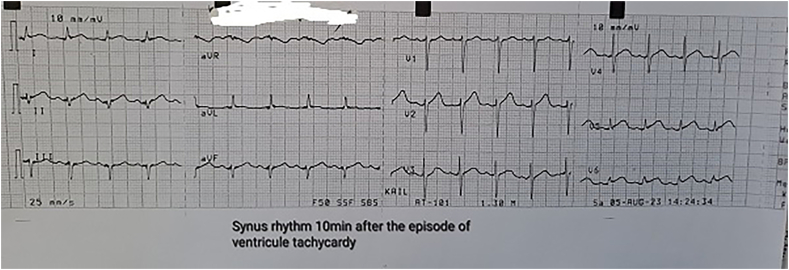
ECG 10minutes after the episode of VT.

**Fig. 5:**
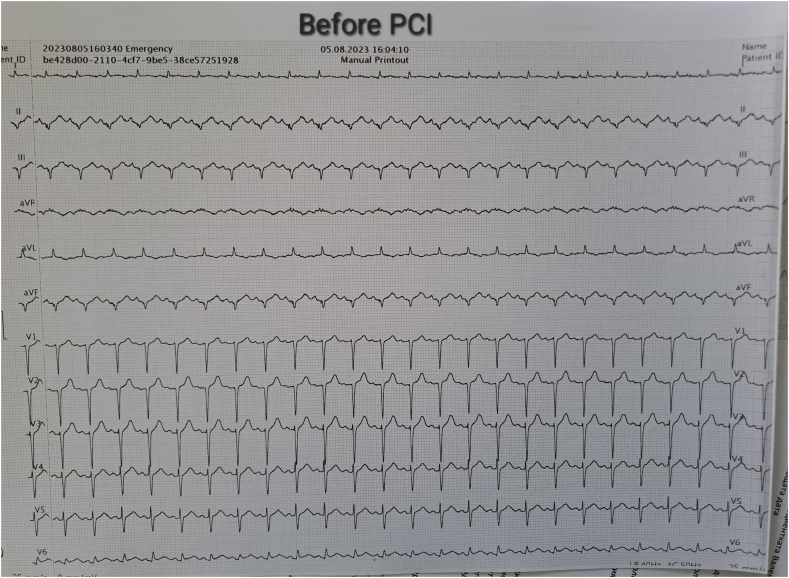
ECG before admission in Cathetarization laboratory for PCI.

**Fig. 6:**
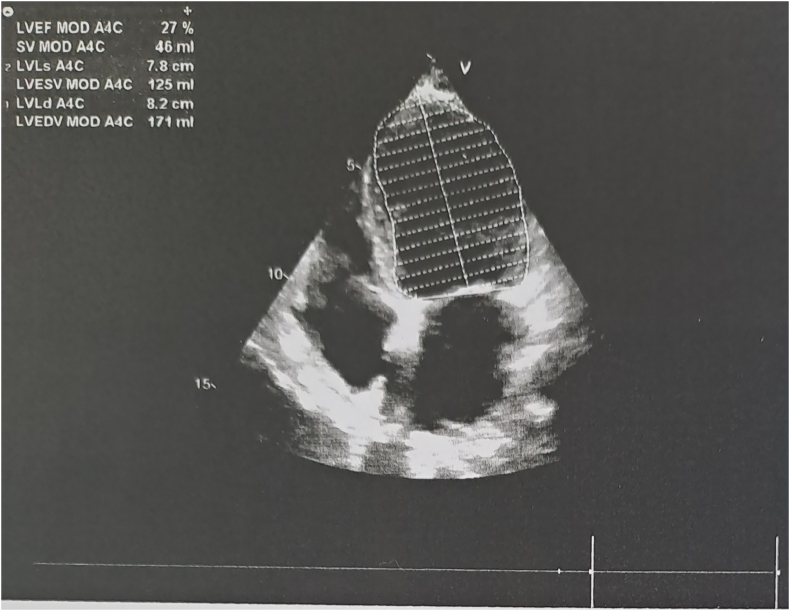
Echogardiography with decreased EF at the beginning of symptoms.

On the first POD, with persistent bleeding from the surgical wound and dynamic hemoglobin values as follow: Hb100g/L, 98g/L, 87g/L, respectively, a wide-complex tachycardia with a ventricular frequency of up to 260/min was recorded on the ECG, which spontaneously passed within a minute and restored sinus rhythm. Amiodarone infusion was started. The patient was conscious, reported a transient feeling of chest pain and inability to breathe. Immediately after the accident hemodynamic measurements reveled mild hypotension - BP 90/50mmHg; sinus tachycardia - HR 120/min; left pathological type of electric axis, elevation of the ST-segment up to 2mm in V2-V6, I, II, negative T-wave in aVL, high sharp symmetrical T-waves on ECG (Fig. 2, Fig. 3, Fig. 4, Fig. 5). An echocardiography revealed severe decreased LV systolic function with an EF of 27% and extensive hypo-akinesia of middle and apical region, preserved kinetics only of the basal segments of the LV (Fig. 6). A coronarography was performed - no coronary artery stenosis.

The main changes in blood tests during the treatment of the patient in the intensive care unit after the manifestations of SC are shown in Table 1.

**Table 1:** Blood tests: thyroid function tests, troponin, CK, CK-MB, BNP.

POD	fT3	fT4	TSH	Troponin	CK	CK-MB	BNP
At accident	3.2	19.4	0.480	0.039	147	100	
1–2 h after accident				0.406	122	22	
2 POD				0.298	136	27	16,132.0
4 POD	2.7	19.3	2.990	0.079	63	14	17,117.0
6 POD		18.2		0.053	36	10	17,178.0
11 POD	2.7	20.8	3.640	0.020	29	19	3446.0

fT3- reference range 3,1–6,8 pmol/L; fT4- reference range 12–22 pmol/L; TSH- reference range 0,27–4,2 mU/L; Troponin- reference range 0–0,014 ng/mL; CK- reference range 0–170 U/L; CK-MB- reference range 0–25 U/L; BNP - reference range, 0–320 pg/mL.

There are no specific treatments for the LV failure characterizing SC. We use upright posture, oxygen therapy, provided by non-invasive ventilation and diuretics for pulmonary pre-edema, amiodarone and magnesium sulfate to treat arrhythmia. The patient received analgesia – combination of Tramadol 100mg and Paracetamol 1.0g three times daily. Anticoagulation was performed as a continues infusion of unfractionated heparin with monitoring of activated partial thromboplastin time (aPTT), aiming levels between 40 and 60s up to 3rd POD, changed to LWMH – Enoxaparine 0.4mL s.c. twice daily. For 72 h after the accident inotropes – Dobutamine in doses 0.5-6μg/kg/min. Mean BP above 60mmHg was maintained with low-dose norepinephrine. Antibiotics, PPI, parenteral/enteral feeding were administrated. Cardiac function was measured by bedside ultrasonography. Subsequently, the patient's condition gradually improved, and she was transferred to the Department of Cardiology for subsequent observation and follow-up.

**Fig. 7:**
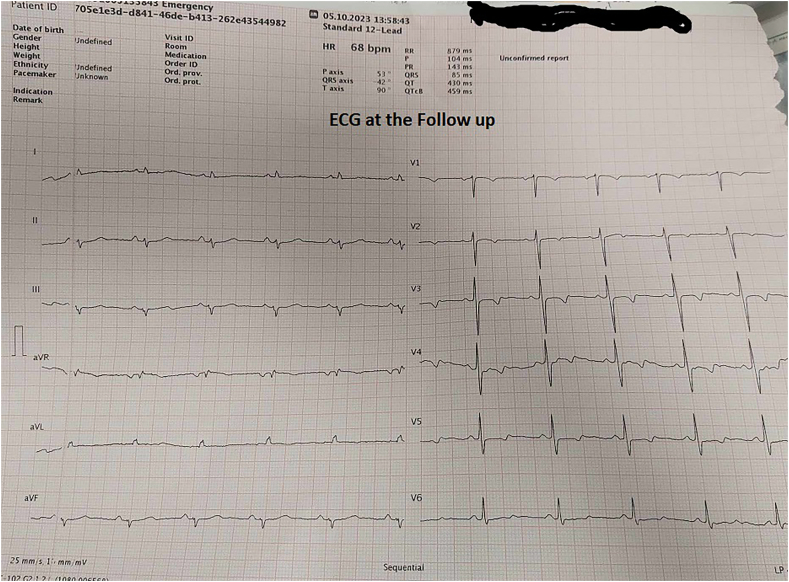
ECG at the follow up.

Two months after the surgery patient underwent through follow-up measuring myocardial function. Echocardiography revealed EF of 57% without any areas of hypokinesia, ECG was normal (Fig. 7). Clinical examination showed no signs of heart failure. We consider the patient was fully recovered.

## 3 Discussion

Stress cardiomyopathy also known as “Takotsubo cardiomyopathy”, “Broken heart syndrome” or “Apical ballooning syndrome”, mimics acute myocardial infarction and it is characterized by acute and fully reversible LV dysfunction. It is frequently caused by emotional or physical stress, and predominantly occurs in postmenopausal women, especially those older than 55 years [1]. SC is an increasingly recognized form of transient LV dysfunction that is often completely reversible [[2], [3], [4], [5]]. It is diagnosed in 1-2% of patients initially presenting with symptoms of acute coronary syndrome - dyspnea, hypotension, syncope, elevated troponin levels, and ST elevations or T wave inversions on electrocardiography [3].

### 3.1 Diagnostic criteria

There is no consensus on the diagnostic criteria for SC. Researchers at the Mayo Clinic proposed diagnostic criteria in 2004, which have been modified recently: 1. transient hypokinesis, akinesis, or dyskinesis in the left ventricular mid segments with/without apical involvement; regional wall motion abnormalities that extend beyond a single epicardial vascular distribution; and frequently, but not always, a stressful trigger; 2. absence of obstructive coronary disease or angiographic evidence of acute plaque rupture; 3. new ECG abnormalities (ST-segment elevation and/or T-wave inversion) or modest elevation in cardiac troponin; and 4. absence of pheochromocytoma and myocarditis [6]. Patients are assigned this diagnosis when they satisfied all these criteria.

### 3.2 Menopause and SC

Stress cardiomyopathy has primarily been described as a disease with a 6-fold female predominance, affecting elderly postmenopausal women leading researchers to an estrogen-based theory for the pathogenesis of this cardiomyopathy [7]. Estrogen modulates vascular tone by upregulating endothelial nitric oxide synthase activity and inhibiting endothelial apoptosis in response to vascular injury. Although estrogen can increase circulating norepinephrine levels, which can result in vasoconstriction, there is net vasodilation in response to increased activity of endothelial nitric oxide synthase. Moreover, women are less sensitive to the hypertensive effects of the renin–angiotensin system as a result of upregulation of type 2 angiotensin II receptors [8]. Estrogen receptors are also expressed in cardiac tissue and can play a role in modulating catecholamine effects on the heart. Meanwhile, within the heart, high circulating levels of norepinephrine and epinephrine, along with increased release and decreased reuptake by sympathetic nerves, induce catecholamine toxicity in the cardiomyocytes via occupation of adrenoreceptors. Hypercontraction and possibly functional basal obstruction of the left ventricular outflow result in increased mechanical wall stress in the LV apex, in conjunction with high BNP levels and increased end-diastolic pressure [9]. Our patient's echocardiography showed similar findings, levels of BNP were extremely high with a peak level at 6-7 POD: 17178,0pg/mL and 17626,0pg/mL, respectively, after that it decreased – 6583,0pg/mL -3446,0pg/mL at 9-10 POD, which was demonstrated by the patient's clinical improvement.

### 3.3 Thyroid disorders and SC

Recently there has been increasing evidence to suggest the occurrence of SC in patients with thyroid dysfunction. It has been reported to occur in patients with Graves' disease, Hashimoto thyroiditis, toxic multinodular goiter, apathetic hyperthyroidism, thyroid storm, iatrogenic hyperthyroidism, subclinical hyperthyroidism, transient hyperthyroid states, following radioactive iodine treatment, following thyroidectomy, and even in hypothyroid or euthyroid states [[10], [11], [12], [13], [14]]. Thyroid hormone increases heart rate and myocardial contractility while simultaneously decreasing systemic vascular resistance, resulting in activation of the renin–angiotensin system. Both genomic and non-genomic actions have been suggested. Triiodothyronine acts at the nuclear level and increases transcriptional activation of cardiac proteins, both structural and regulatory proteins such as Na^+^/K^+^ ATPase, sarcoplasmic reticulum Ca^2+^-ATPase, voltage-gated ion channels, myosin heavy chain and β-adrenergic receptors. Non-genomic effects involve membrane ion channels and endothelial nitric oxide synthase, resulting in vascular relaxation and decreased systemic resistance [15]. SC has also been documented to occur in patients with hypothyroid or subclinical hypothyroid states [16]. However, the mechanism by which different thyroid functional states can trigger SC remains unclear.

Although the patient in the present case was euthyroid both preoperatively and in the early postoperative period, the large mass of the thyroid gland combined with the postmenopausal syndrome, the operative stress and the stress, added by the bleeding in the early postoperative period in combination, are probably the reason for the development of SC.

## 4 Conclusion

In this case, the clinical, laboratory course and echocardiography showed similar findings, so SC was strongly suspected. Coronary angiography and left ventriculography confirmed the diagnosis. The patient had a good course and was discharged on the 14 POD (13 day after accident of SC) hemodynamically stable, with good control of BP and heart rate, with improved kinetics - EF 46%, compared to the previous echocardiography. If cardiovascular complications are observed during the perioperative period for thyroid gland surgery, SC should be kept in mind especially in women with postmenopausal syndrome.

## 5 Methods

The work has been reported in line with the SCARE criteria [17].

## Footnotes

Not applicable.

## Consent for publication

Written informed consent was obtained from the patient for publication of this case report and accompanying images. A copy of the written consent is available for review by the Editor-in-Chief of this journal on request.

## Availability of data and materials

Not applicable.

## Ethical approval

We consider that ethical approval is not required because our case report is not a study, we did not conduct any off-label or new treatment or investigation. We report an unusual complication that might be taken in mind in this situation.

## Funding

No funding was used.

## Author contribution

Margarita Atanasova - Writing - original draft; Conceptualization; Resources

Manol Sokolov Writing - review & editing

Tsvetan Popov - Writing - review & editing, Data curation; Resources

Angel Arabadzhiev - Writing - review & editing, Data curation;

Silvia Ivanova - Writing - review & editing, Data curation;

Gergana Ivanova - Writing - original draft; Methodology; Resources

All authors read and approved the final manuscript.

## Guarantor

Gergana Ivanova, MD.

## Research registration number

1.Name of the registry: Not applicable.

2.Unique identifying number or registration ID: Not applicable.

3.Hyperlink to your specific registration (must be publicly accessible and will be checked): Not applicable.

## Conflict of interest statement

The authors declare that they have no competing interests.
